# Supplementary figures and images for: Exercise and Sestrin Mediate Speed and Lysosomal Activity in Drosophila by Partially Overlapping Mechanisms
Source: Cells. 2021 Sep 19;10(9):2479. doi: 10.3390/cells10092479 (PMC8466685; doi:10.3390/cells10092479)

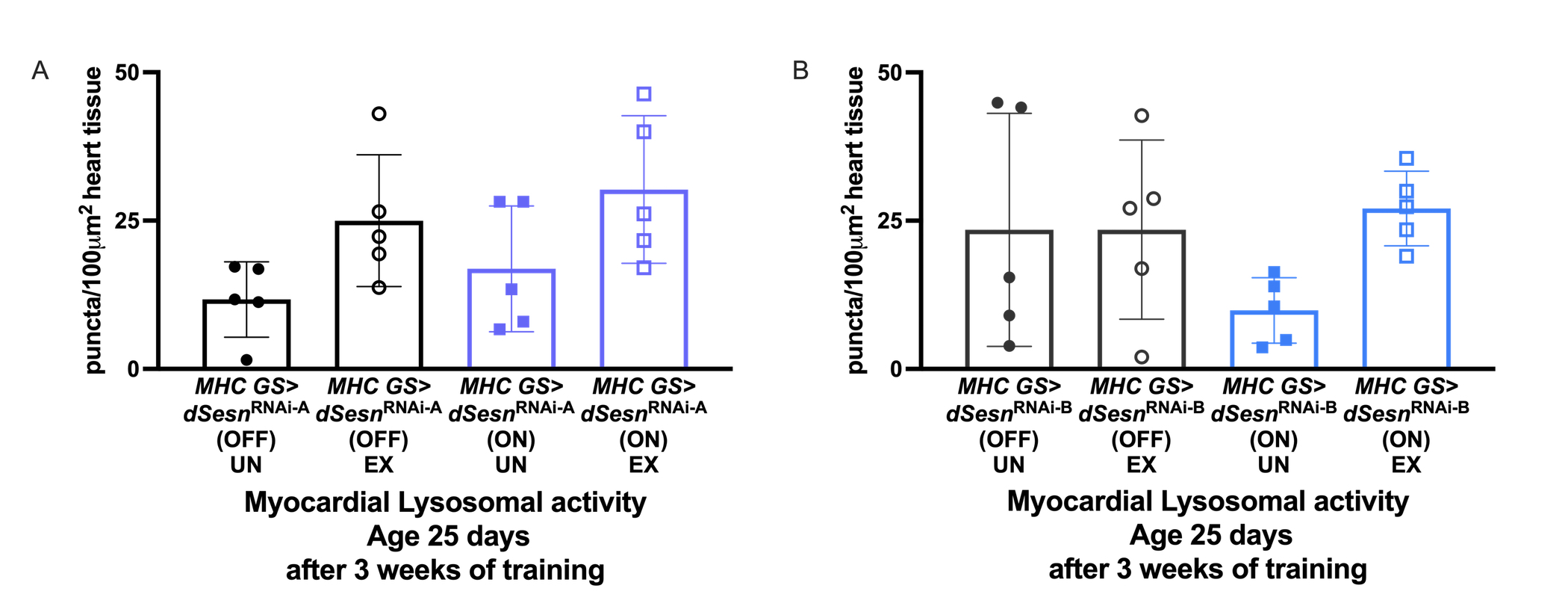

Supplement: Supplementary file 1 [file cells-10-02479-s001.zip › cells-1366339-suppl/Supplemental Figure S1.jpg]
